# CRISPR screen identifies *CEBPB* as contributor to dyskeratosis congenita fibroblast senescence via augmented inflammatory gene response

**DOI:** 10.1093/g3journal/jkad207

**Published:** 2023-09-17

**Authors:** Erik R Westin, Alireza Khodadadi-Jamayran, Linh K Pham, Moon Ley Tung, Frederick D Goldman

**Affiliations:** Department of Pediatrics, Division of Hematology Oncology, University of Alabama at Birmingham, Birmingham, AL 35294, USA; Department of Cancer Precision Medicine, Pennington Biomedical Research Center, Louisiana State University, Baton Rouge, LA 70808, USA; Genome Technology Center, Applied Bioinformatics Laboratories, NYU Langone Medical Center, New York, NY 10016, USA; Department of Pediatrics, Division of Hematology Oncology, University of Alabama at Birmingham, Birmingham, AL 35294, USA; Stead Family Department of Pediatrics, Division of Medical Genetics and Genomics, University of Iowa, Iowa City, IA 52242, USA; Department of Pediatrics, Division of Hematology Oncology, University of Alabama at Birmingham, Birmingham, AL 35294, USA

**Keywords:** Dyskeratosis congenita, telomere, oxidative stress, inflammatory response, CRISPR, human primary fibroblasts, *CEBPB*, *WSB1*, *MED28*, *p73*

## Abstract

Aging is the consequence of intra- and extracellular events that promote cellular senescence. Dyskeratosis congenita (DC) is an example of a premature aging disorder caused by underlying telomere/telomerase-related mutations. Cells from these patients offer an opportunity to study telomere-related aging and senescence. Our previous work has found that telomere shortening stimulates DNA damage responses (DDRs) and increases reactive oxygen species (ROS), thereby promoting entry into senescence. This work also found that telomere elongation via TERT expression, the catalytic component of the telomere-elongating enzyme telomerase, or p53 shRNA could decrease ROS by disrupting this telomere–DDR–ROS pathway. To further characterize this pathway, we performed a CRISPR/Cas9 knockout screen to identify genes that extend life span in DC cells. Of the cellular clones isolated due to increased life span, 34% had a guide RNA (gRNA) targeting *CEBPB*, while gRNAs targeting *WSB1*, *MED28*, and *p73* were observed multiple times. *CEBPB* is a transcription factor associated with activation of proinflammatory response genes suggesting that inflammation may be present in DC cells. The inflammatory response was investigated using RNA sequencing to compare DC and control cells. Expression of inflammatory genes was found to be significantly elevated (*P* < 0.0001) in addition to a key subset of these inflammation-related genes [*IL1B*, *IL6*, *IL8*, *IL12A*, *CXCL1* (*GROa*), *CXCL2* (*GROb*), and *CXCL5*]. which are regulated by CEBPB. Exogenous *TERT* expression led to downregulation of RNA/protein CEBPB expression and the inflammatory response genes suggesting a telomere length-dependent mechanism to regulate CEBPB. Furthermore, unlike exogenous TERT and p53 shRNA, *CEBPB* shRNA did not significantly decrease ROS suggesting that *CEBPB's* contribution in DC cells’ senescence is ROS independent. Our findings demonstrate a key role for *CEBPB* in engaging senescence by mobilizing an inflammatory response within DC cells.

## Introduction

Senescence is the result of cellular programming to prevent aged, damaged, or abnormal cells from continued proliferation. Cell intrinsic/extrinsic factors include telomere attrition (due to steady-state proliferation), DNA damage (oxidative stress, UV, etc.), and oncogene-induced senescence (e.g. RAS^V12^). These pathways can alter gene expression and arrest the cell cycle [e.g. senescence-associated secretory phenotype (SASP), p16^INK4A^].

Elongated telomeres suppress senescence by forming a stable secondary structure called a T-loop that prevents activation of the double-strand DNA break (DSB) repair pathway (d'Adda di Fagagna *et al*. 2003; Griffith *et al*. 1999; de Lange 2018). However, systematic telomere attrition leads to critically shortened telomeres thus initiating a DSB response. This DSB pathway involves activation of ATM/ATR followed by p53 to arrest the cell cycle via various p53-responsive genes including the cyclin-dependent kinase inhibitor p21^CDKN1A^ (Stein *et al*. 1999; Engin and Engin 2021). Sustained telomere dysfunction leads to replicative senescence via p53-p21^CDKN1A^ in addition to p16^INK4A^-RB (d'Adda di Fagagna *et al*. 2003; Smogorzewska and de Lange 2002). Furthermore, the SASP pathway can promote senescence to neighboring cells via paracrine/autocrine effects by releasing proinflammatory cytokines, chemokines, and growth factors within a niche (Acosta *et al*. 2013; Kuilman *et al*. 2008; Lopes-Paciencia *et al*. 2019). SASP alters the local environment through extracellular matrix remodeling and is stimulated independent of p53 and p16 activation (Coppé *et al*. 2011; Rodier *et al*. 2009).

Dyskeratosis congenita (DC) is characterized by telomere shortening due to mutations in telomerase- and telomere-related genes. A partial list of these genes includes *TERC* (H/ACA box-containing noncoding RNA template; component of the telomere-elongating enzyme, telomerase), *TERT* (protein/catalytic component of telomerase), *DKC1* (dyskerin protein, binds H/ACA RNAs), and *TINF2* (telomere-stabilizing protein interacts with telomere-binding proteins TRF1 and TRF2). The clinical DC phenotype is variable and classically consists of leukoplakia, abnormal skin pigmentation, and nail dystrophy in addition to pulmonary fibrosis and bone marrow failure. While hematopoietic stem cell (HSC) transplantation is the only curative therapy for bone marrow failure in DC, it is associated with significant posttransplant complications. Using several different cell types, including HSCs, fibroblasts, and lymphocytes from varying DC genotypes, we have found these cells display premature DNA damage responses (DDRs), resulting in elevated reactive oxygen species (ROS) (Pereboeva *et al*. 2016, 2013; Sorge *et al*. 2017; Westin *et al*. 2011, 2007). The increase in ROS appears to be related to telomere shortening/p53 activation; experimentally elongating telomeres or disrupting p53 led to a concomitant decrease in ROS and an increased cellular life span (Westin *et al*. 2011).

One strategy to better understand a pathway like telomere-dependent senescence is to deploy a genome-wide screen. The GeCKO (genome-scale CRISPR-Cas9 knockout) lentiviral library delivers CRISPR/Cas9 to cut gRNA-targeted genes, knocking them out via insertions/deletions created by nonhomologous end joining (NHEJ) (Shalem *et al*. 2014).

We designed a forward genetic experiment whereby primary fibroblasts from a DC patient (*TINF2* mutation) were transduced with the GeCKO library. We identified a number of candidate genes, including the transcription factor *CEBPB*, that regulate senescence in telomere-shortened cells. RNA sequencing (RNA-seq) revealed a significant inflammatory gene expression response (*IL1B*, *IL6*, *IL8*, *IL12A*, *CXCL1* (*GROa*), *CXCL2* (*GROb*), and *CXCL5*) in DC cells that could be corrected upon exogenous *TERT* (exogTERT) expression. Cells expressing exogTERT also had a decrease of both CEBPB (RNA and protein) and CEBPB–SASP-related gene expression. These findings identify new genes and downstream pathways that could promote senescence in a background of shortened telomeres.

## Materials and methods

### Cell culture

This study was approved by the University of Alabama at Birmingham Internal Review Board (F100512004). Skin punch biopsies were collected from DC patients and healthy volunteers after written informed consent. DC mutations included *TINF2* (R282C) and *TERT (*R631W) as previously reported (Pereboeva *et al*. 2016) in addition to *DKC1* (A353V). Primary skin fibroblasts were expanded and grown in DMEM supplemented with 10% fetal bovine serum under atmospheric oxygen conditions, as previously described (Westin *et al*. 2011). Fibroblast colonies requiring cloning were ring-cloned to permit characterization of gRNAs expressed. To this end, sterile silicone grease (Fisher Science) was applied to the bottom of sterile cloning cylinders (Fisher Science). These cloning cylinders were adhered to 10-cm cell culture plates to seal off clones from other cells. Media within cylinder was aspirated and cells PBS washed and trypsinized. Clones were further expanded in 24-well and 6-well plates to permit DNA isolation.

### Lentivirus production/cell transduction

The GeCKO lentiviral virus library, designed by the Zhang lab (Shalem *et al*. 2014), was acquired from Addgene (https://www.addgene.org/pooled-library/zhang-human-gecko-v2/). DNA purification adjustments were made to ensure equal amplification of each DNA vector (∼120 k, ∼6× coverage of each gene) (Shalem *et al*. 2014; https://media.addgene.org/cms/files/GeCKOv2.0_library_amplification_protocol.pdf). For lentiviral virus production {GeCKO, CEBPB/p53/scrambled shRNA [(Dharmacon)], 293 T HEK cells were transfected with the purified DNA library or vector in combination with accessory lentiviral plasmids (VSVG, Rev, and Gag/Pol) using Fugene 6 (Promega}. Cells were washed on day 1 to remove DNA/Fugene 6 and supernatants collected on days 2 and 3. Approximately 1 × 10^6^ cells were transduced with lentiviral supernatants at an MOI of ∼1 in the presence of polybrene (4 µg/mL) during log-phase growth and washed 16 h later. Puromycin antibiotic selection (1 µg/mL) was added to media for 5 days to isolate cells infected with virus. To isolate gRNA clones derived from the GeCKO library transductions, cell colonies were ring-cloned to permit sequencing gRNAs. Retroviral exogenous TERT (Westin *et al*. 2007) was produced with methodology similar to lentivirus production; however, 293T Phoenix cells expressing Gag/Pol (National Gene Vector Biorepository) were used and transfected with pVSVG.

### DNA cloning

DNA was procured from ring-cloned colonies using QIAquick DNA extraction kit (Qiagen). Isolated genomic DNA was PCR-amplified (Ex Taq, Takara) with primers flanking the gRNA common to each GeCKO. PCR-amplified DNA was cloned using the TA cloning system (Invitrogen) and transformed into DH5α bacteria. Individual bacterial colonies were isolated, DNA-extracted, and submitted for Sanger sequencing (4× bacterial colonies per cellular clone).

### RNA-seq

Midpassage primary DC (TINF2, DKC1, and TERT) and control fibroblasts were scraped in the presence of Trizol. RNA was extracted using the Qiagen RNeasy Mini RNA extraction kit. Total RNA was submitted for sequencing using the NextSeq500 (Illumina). Sequencing reads were mapped to the reference genome (hg19) using the STAR aligner (v2.5.0c) (Dobin *et al*. 2013). Alignments were guided by a Gene Transfer Format (GTF) file. The mean read insert sizes and their standard deviations were calculated using Picard tools (v.1.126) (http://broadinstitute.github.io/picard). The read count tables were generated using HTSeq (v0.6.0) (Anders, Pyl, and Huber 2015), normalized based on their library size factors using DEseq2 (Love, Huber, and Anders 2014), and differential expression analysis was performed. The reads per million (RPM) normalized BigWig files were generated using BEDTools (v2.17.0) (Quinlan and Hall 2010) and bedGraphToBigWig tool (v4). KEGG pathway analysis and gene ontology (GO) analysis were performed using the clusterProfiler R package (v3.0.0) (Yu *et al*. 2012) and gene set enrichment analysis (GSEA) was performed using GSEA (Mootha *et al*. 2003; Subramanian *et al*. 2005).

### Oxidative stress

ROS analysis was performed as previously reported (Westin *et al*. 2011). Briefly, for FACS-based dihydroethidium (DHE, Sigma) ROS detection, fibroblasts were incubated in the presence of 10 µM DHE/PBS/2% FBS/5 mM sodium pyruvate for 15 min at 37°C. After 2× PBS washing, cells were trypsinized and placed on ice. ROS levels were quantified by recording the mean fluorescent intensity (MFI). As a positive control, antimycin A (10 µM, Sigma) was used to stimulate ROS production.

### qRT-PCR

Expression of CEBPB was examined in cells expressing 5 different CEBPB shRNAs (Dharmacon). Isolated RNA was converted to cDNA via iScript cDNA synthesis (Bio-Rad). CEBPB and β-actin (ACTB gene) were amplified from cDNA with Power SYBR Green PCR Master Mix (Life Tech) to assess relative expression between control (scrambled shRNA) and CEBPB shRNA cells. Analysis was performed on the ViiA Real-Time PCR expression analyzer (Life Tech) using equation 2^−ΔΔCT^ (Livak and Schmittgen 2001).

### Western blotting

Standard western blotting techniques were used as previously described (Pereboeva *et al*. 2016). Briefly, cells were pelleted and lysed with complete Lysis-M buffer (Roche). Whole-cell extracts (20,000 cells) were subjected to SDS-PAGE electrophoresis, transferred to a nitrocellulose membrane, and stained with C/EBPβ-HRP (1:1,000) and β-actin (1:500) antibodies (Santa Cruz); β-actin was followed by rabbit antimouse-HRP (Santa Cruz). Signal was visualized post-ECL Prime Western Blotting Detection Reagent staining (GE).

### Statistical analyses

Statistical significance was evaluated using the Student's *t*-test between 2 groups of data under the null hypothesis and reported by calculated *P*-values. Error bars found within graphs illustrate the standard deviation of DC or control samples in each experiment.

## Results

### CRISPR knockout screen

**Fig. 1.**
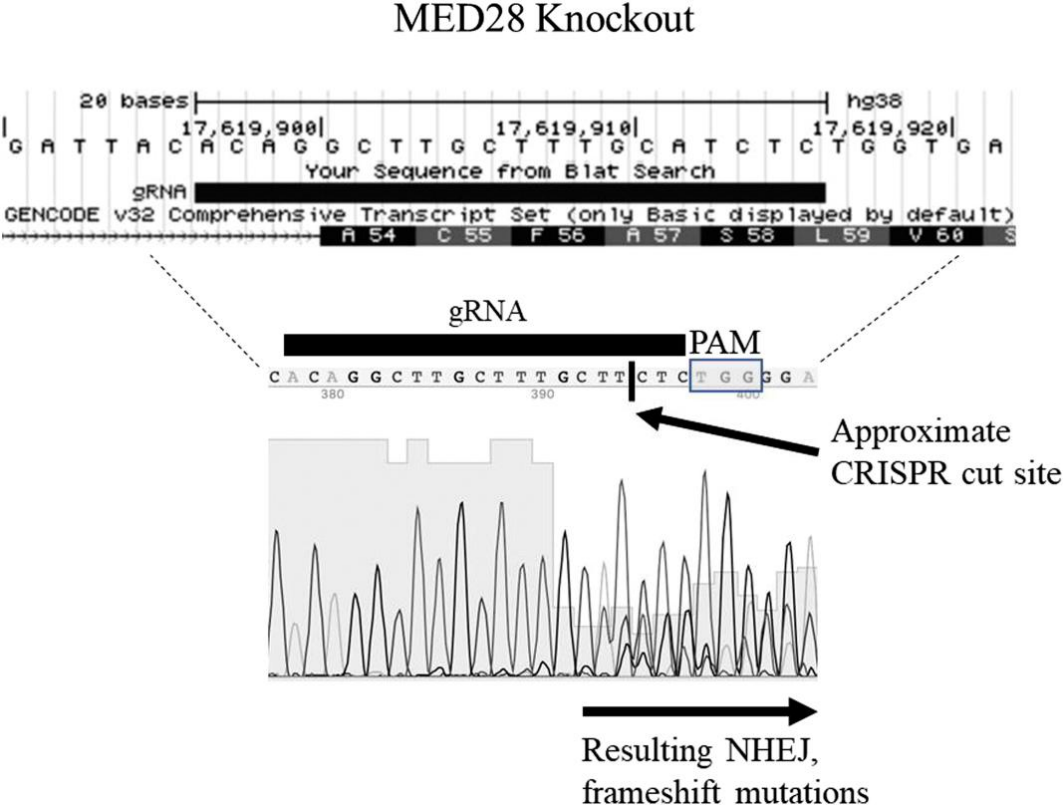
Characterization/validation of knockout library. Gene diagram (UCSC Genome Browser, https://genome.ucsc.edu/index.html) indicating location of 20-bp gRNA within MED28 gene. Approximate cut site of MED28 gene knockout 3-bp upstream of protospacer adjacent motif (PAM). Variable Sanger sequence downstream of cut site a result from frameshift mutations found within clone.

We set out to identify human genes that promote senescence due to telomere dysfunction using primary skin fibroblasts from a DC patient with a single *TINF2* mutation (R282C). *TINF2*-mutated fibroblasts were transduced with the GeCKO lentiviral CRISPR knockout library at a low multiplicity of infection (MOI) to prevent transduction of cells with multiple viruses/gRNAs. In total, 38 colonies were isolated and submitted for Sanger sequencing of the incorporated gRNAs, uncovering ∼42 gRNAs with an average 2.0 unique gRNAs per colony. To verify that the gRNAs were indeed cutting the locus of interest, genomic DNA was PCR-amplified flanking the cut site and Sanger-sequenced to verify genomic targeting. A representative figure of typical CRISPR verification is presented in Fig. 1. Sequencing of a single MED28 clone revealed an insertion/deletion (indel) at the site targeted by the gRNA. Sequencing of a CEBPB clone uncovered a 600-bp exonic deletion on 1 allele, and the inability to PCR amplify the other allele suggests a larger deletion encompassing the primers within this region. In addition to *CEBPB* and *MED28*, 2 other genes were also found repeatedly knocked out and deemed high-value. As noted in Table 1, these included gRNAs for *CEBPB* (13 clones), *WSB1* (3 clones), *MED28* (3 clones), and *TP73* (3 clones).

**Table 1.** Key genes identified from knockout library.

Gene KO	Total gRNA clones (*n* = 38)	Clones with single gRNA (*n* = 11)	gRNA sequence (5′ to PAM)	Protein classification
CEBPB	13	8	TACGCGCTGCGCGCTTACCT	Gene-specific transcriptional regulator
WSB1	3	1	CAGTATGATCTACCAAGTTA	WD repeat and SOCS box, E3 ubiquitin ligase complex
MED28	3	1	ACAGGCTTGCTTTGCATCTC	Mediator of RNA polymerase II transcription
TP73	3	1	GCGGGGTGGACACCTTGATC	Likely tumor suppressor; gene-specific transcriptional regulator

Characterization of gene knockouts from 38 cellular clones found multiple occurrences of the same gene. For example, CEBPB was found 13 times within 38 clones, 8 times of which it was the only gRNA uncovered (8 clones with single gRNA + 5 clones with CEBPB/addition gRNA(s) = 13).

### CEBPB and inflammatory responses

**Fig. 2.**
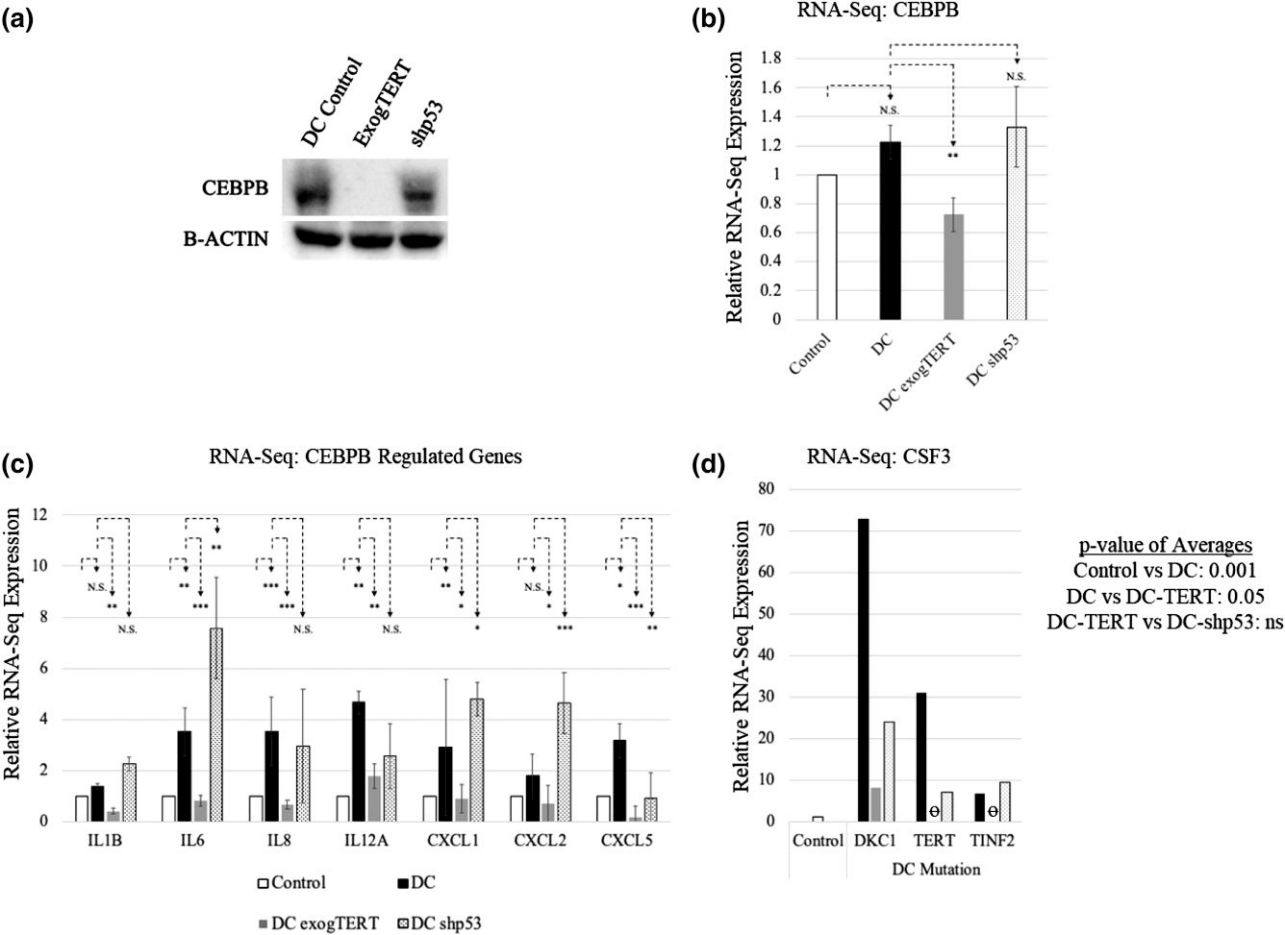
Exogenous TERT decreases CEBPB protein and CEBPB-related SASP. a) CEBPB protein expression decreased upon TERT expression but not p53 shRNA, in TINF2-mutated DC fibroblasts. b) RNA-seq expression of CEBPB in unmodified, TERT-expressing (exogTERT), and shp53-expressing DKC1-, TERT-, and TINF2-mutated fibroblasts. c) Targeted analysis of CEBPB-regulated genes from RNA-seq. d) RNA-seq expression of CSF3 (G-CSF) within each DC genotype and related experimental modifications (control vs DC: *P* < 0.001; DC vs DC exogTERT: *P* < 0.05; DC vs DC-shp53: not statistically significant). Statistical significance was determined based on comparisons between (1) control and DC cells (TINF2, DKC1, and TERT cells), (2) DC cells and DC exogTERT cells (all genotypes), and (3) DC and DC shp53 expressing cells (all genotypes). Data normalized to control expression; error bars represent the standard deviation of gene expression among all 3 mutated genotypes. Student's *t*-test: *P < 0.05, **P < 0.01, and ***P < 0.001; N.S., not significant. “o” signifies no expression.

The knockout screen provided evidence that short telomeres activate CEBPB to promote senescence in primary DC cells. Past research by our lab demonstrated DC senescence could be overcome upon telomere elongation by exogTERT or p53 knockdown (Westin *et al*. 2011). We tested whether protein levels of CEBPB were altered in DC whole-cell lysates when expressing either exogTERT or *p53* shRNA in *TINF2*-mutated cells. Expression of exogTERT, but not *p53* shRNA, caused near complete loss of CEBPB protein expression (Fig. 2a). That *p53* knockdown did not reduce CEBPB suggests that upregulation of CEBPB in DC cells may occur independent of the DDR pathway.

CEBPB and CEBPB-regulated genes have been associated with a proinflammatory response and SASP (Salotti and Johnson 2019). To investigate whether CEBPB-regulated genes were altered in primary DC cells, we performed RNA-seq analyses on DC fibroblasts with different genotypes (*DKC1*, *TINF2*, and *TERT*). Each genotype was transduced with vector control, exogTERT or *p53* shRNA, and examined for gene expression changes by pooling genotypes together. DC cells expressing exogTERT showed a 40% decrease in CEBPB expression (*P* < 0.01; Fig. 2b) but not shp53. A significant increase in SASP-related, CEBPB-dependent genes were found in primary DC cells compared to controls (white vs black bars; Fig. 2c). Furthermore, this inflammatory profile was downregulated with exogTERT (black vs gray bars). Of interest, the inflammatory response profile persisted when *p53* shRNA was expressed (black vs dotted bars). Expression of another SASP factor, CSF3 (G-CSF), was the most variable among all genotypes in the targeted analysis of CEBPB-regulated genes. Its expression among the 3 DC genotypes averaged a 37-fold increase (*P* < 0.001) and decreased an average of ∼20× in exogTERT cells (*P* < 0.05; Fig. 2d).

**Fig. 3.**
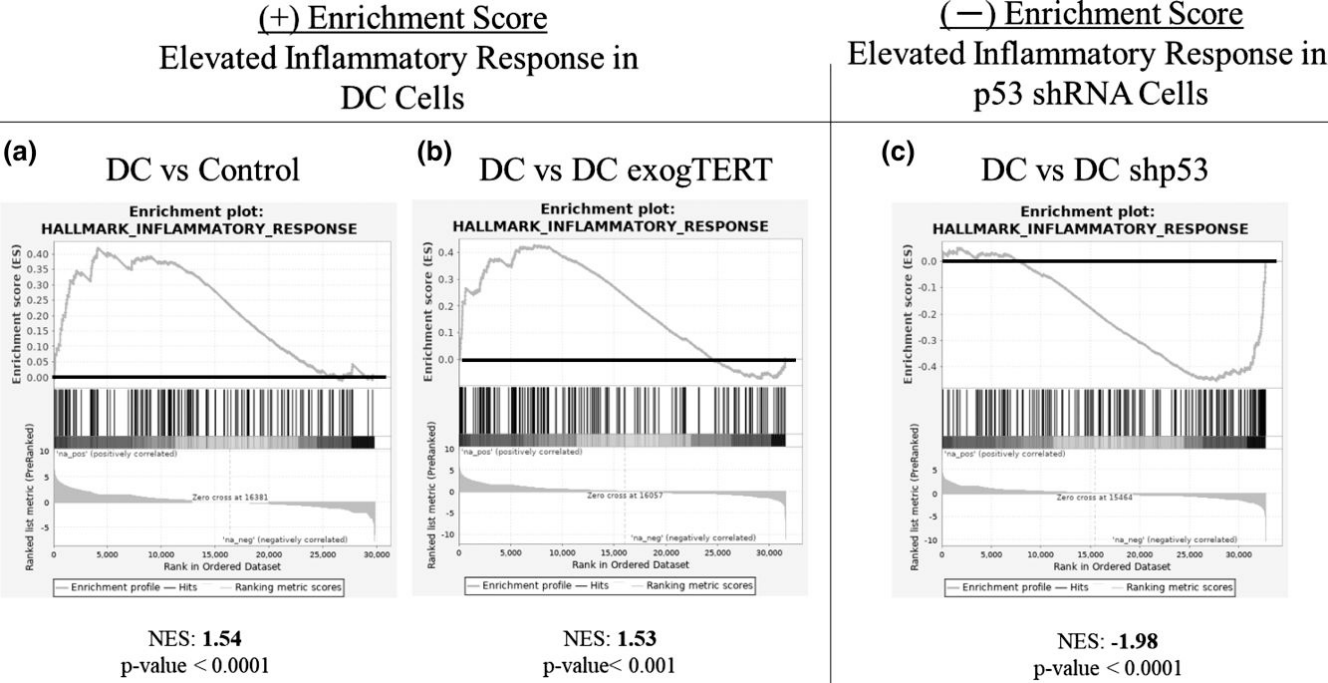
Elevated inflammatory response in DC cells, suppressed DC cells expressing exogenous TERT. RNA-seq analysis provided expression changes to perform GSEA (Mootha *et al*. 2003, Subramanian *et al*. 2005) on control and DC skin fibroblasts. The enrichment score analysis presents the ranked order of genes from overexpressed (left) to underexpressed (right). The more or less enriched a particular gene set is compared to a control set, the more positive/negative the normalized enrichment score (NES) and thus the greater the *P*-value. DC samples from patients with 3 different genotypes (TINF2, DKC1, and TERT mutations) were individually analyzed and averaged to determine a “DC expression signature”. a) DC cells had a positive enrichment score for inflammatory response when compared to control or b) TERT-expressing DC fibroblasts (exogTERT; NES 1.54/*P* < 0.0001 and NES 1.53/*P* < 0.001 respectively, also from TINF2, DKC1, and TERT mutations). c) DC cells had a negative enrichment score when compared to p53 shRNA-expressing DC cells (NES −1.98/*P* < 0.0001, all 3 genotypes).

GSEA was performed on the RNA-seq data sets and provided further evidence that primary DC cells have a significant underlying inflammatory response (Fig. 3a; positive enrichment score: 1.54, *P* < 0.0001). Using all 3 genotypes, a pairwise comparison of DC cells and those expressing exogTERT found a statistically significant difference (Fig. 3b; positive enrichment score: 1.53, *P* < 0.0001). However, knocking down *p53* in DC cells created a more robust inflammatory response than untreated DC cells (Fig. 3c; negative enrichment score: −1.98, *P* < 0.0001). Together these data suggest that DC cells mobilize CEBPB to augment the expression of SASP-related genes to promote an inflammatory response. The concomitant decrease in CEBPB gene/protein expression and genes regulated by CEBPB in exogTERT cells suggests the reduced SASP/inflammatory response is likely telomere length-dependent.

### CEBPB knockdown does not impact ROS

**Fig. 4.**
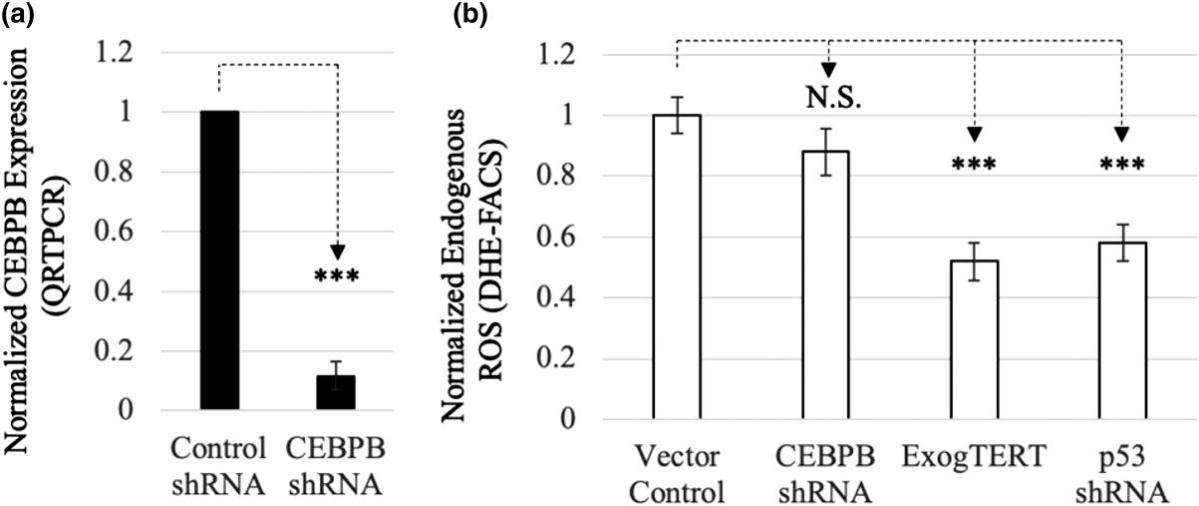
CEBPB downregulation does not impact ROS. TINF2-mutated DC cells were infected with 5 different CEBPB shRNAs and examined for an expression decrease and presence of ROS (representative shRNA knockdown presented in figure). a) Expression of CEBPB was determined by qRT-PCR (8.5× decrease bars). b) ROS levels were examined by staining cells with dihydroethidium (DHE) and quantified by FACS analysis using the MFI. Exogenous TERT (exogTERT) and p53 shRNA-expressing DC cells provided as controls to compare to CEBPB shRNA-expressing cells. Levels were normalized to the empty vector control. Significance was determined by Student's *t*-test; ***P < 0.001; N.S.: not significant.

Telomere elongation or *p53* knockdown can both extend the life span of primary DC cells and decrease ROS levels (Westin *et al*. 2011). We hypothesized that if CEBPB was integral to the short telomere–p53–ROS pathway, then decreasing *CEBPB* expression would also reduce ROS. We expressed *CEBPB* shRNA in DC cells and analyzed ROS using DHE staining and FACS analysis. As noted in Fig. 4a, although we found a significant decrease in shRNA-targeted *CEBPB* expression by qRT-PCR (83% reduction, *P* < 0.001), there was no significant decrease in ROS within these same cells (Fig. 4b). Decreases in ROS could be found in both DC-exogTERT and DC-shp53-expressing cells (*P* < 0.001), as previously reported. These results provide evidence that primary DC cells may have multiple, redundant, prosenescence pathways that are either DDR/ROS-dependent or CEBPB-dependent.

## Discussion

The mechanism by which telomere shortening promotes senescence is not entirely understood. DC, a well-described telomere biology disorder, consists of pathologies related to premature aging/cellular senescence. Causative DC mutations are found in genes related to telomere stability and maintenance (Mason and Bessler 2011; Tummala *et al*. 2015; Dokal *et al*. 2015; Revy, Kannengiesser, and Bertuch 2023). Since telomere dysfunction is the critical factor in promoting senescence, DC cells are useful to study this relationship. We have previously found proliferative defects, activated DDR, and increased ROS in several cell types and mutations from DC patients (Pereboeva *et al*. 2016, 2013; Sorge *et al*. 2017; Westin *et al*. 2011, 2007). We showed that shortened telomeres activate DDR/p53 leading to increased ROS. In addition, telomere elongation (exogTERT) or blocking DDR (*p53* shRNA) rescued proliferation and decreased ROS (Westin *et al*. 2011).

The GeCKO library provided a tool to systematically knock out genes in DC cells to recover those critical for senescence. The *CEBPB* gRNA repeatedly appeared in this CRISPR KO screen, signifying its importance (Table 1). CEBPB is a member of a family of CEBP proteins that contain a basic leucine zipper required for binding and initiating/repressing gene transcription (Ramji and Foka 2002). CEBPB is well characterized and key to immune/inflammatory responses and when overexpressed has potent cytostatic activity (Johnson 2005; Freund *et al*. 2010). CEBPB has a known role in regulating genes involved in the inflammatory response [e.g. *IL6*, *IL8*, *IL1B*, *IL12A*, *CXCL1* (*GROa*), *CXCL2* (*GROb*), *CXCL5*, *CSF3* (*G-CSF*)] and others (Akira *et al*. 1990; van der Krieken *et al*. 2015; Salotti and Johnson 2019; Freund *et al*. 2010). Our data also found CEBPB RNA and protein expression were suppressed in response to exogTERT. This could provide an explanation and a link for the concomitant suppression of the underlying inflammatory response in DC cells (Fig. 2a). Our previous research identified that both DDRs and ROS respond to telomere length (Westin *et al*. 2011). This previous research found (1) DDR and ROS were ameliorated with telomere elongation, bypassing senescence, and (2) ROS could be decreased by blocking p53 signaling in a background of shortened telomeres (also bypassing senescence). This allowed the design of a simple hypothesis: shortened telomeres increase DDR and therefore ROS to promote and enforce senescence. In this research, we have amended our hypothesis with the finding that telomere elongation decreases inflammatory response, independent of DDR and ROS. These data suggest that (1) CEBPB functionality lies downstream of telomere signaling, but (2) CEBPB activity is independent of p53 activation. Finally, the fact that similar RNA expression was found among all DC genotypes, including with exogTERT and shp53 expression, suggests that CEBPB and the inflammatory phenotype are telomere length-dependent and independent of DC genotype. Aging-related knockout screens have also been performed elsewhere but did recover CEBPB (Liu *et al*. 2019). This screen was designed a priori to knockout genes related to senescence, however, and it is not clear if CEBPB was part of the screen. Consistent with our findings, this screen did find the involvement of inflammation. The proinflammatory SASP includes gene and protein expression changes of secreted proteins (i.e. growth factors, interleukins, and chemokines) that are responsible for alterations in the local microenvironment through paracrine effects (Coppé *et al*. 2010). Another study examined 9 cytokine levels in serum of patients diagnosed with inherited bone marrow failure disorders. They found that, in general, these proteins were not elevated in DC patients (Giri *et al*. 2015). However, upon stratification of DC patients based on the presence of severe bone marrow failure, significant levels of CSF3 (G-CSF) were found, consistent with our results (Fig. 2d). The presence of an intrinsically upregulated inflammatory response suggests that current antiinflammatory approaches could be repurposed to attenuate disease pathologies found in DC patients.

The GeCKO screen also recovered a gRNAs targeting *MED28*, *p73*, and *WSB1* gRNAs. *MED28* is a subunit of the Mediator complex, a large ∼26 submit complex that regulates transcription. Mediator acts as a bridge between the activation domains of transcription factors and RNA polymerase II in response to intracellular signaling (Allen and Taatjes 2015). CEBPB differentially binds the Mediator complex depending on whether it is transcriptionally active (Mo *et al*. 2004), suggesting a common mechanism for *CEBPB* and *MED28* knockouts. *p73* is a homolog to *p53* and has significant sequence and functional overlap. p73 can bind canonical p53 binding sites, oligomerize with p53 protein (Dötsch *et al*. 2010), and induce cell-cycle arrest, apoptosis, and senescence (Dötsch *et al*. 2010). Based on our previous experiments using *p53* shRNA, we predicted knockout of *p53* would be recovered in this screen. Of note, 1 clone included a p53-targeting gRNA (data not shown) confirming the capacity of the screen to return credible gene candidates. Finally, knockout of WSB1 may have imparted and extended life span via an E3 ubiquitin ligase that targets and destabilizes ATM through ubiquitination in 293T and MEF cells (Kim *et al*. 2017).

We cannot rule out that alternative explanations may account for increased life span in colony knockouts. Gene knockouts may have led to the activation of endogenous *TERT* expression or blocked a functional DDR from taking place. It is also possible that gRNAs may have targeted and knocked out unanticipated genes; however, off-target minimization was a consideration designing the library (Shalem *et al*. 2014). Each gRNA had possible off-targets though no off-target sequence had 100% homology to the gRNA. These alternative explanations will require further investigation.

**Fig. 5.**
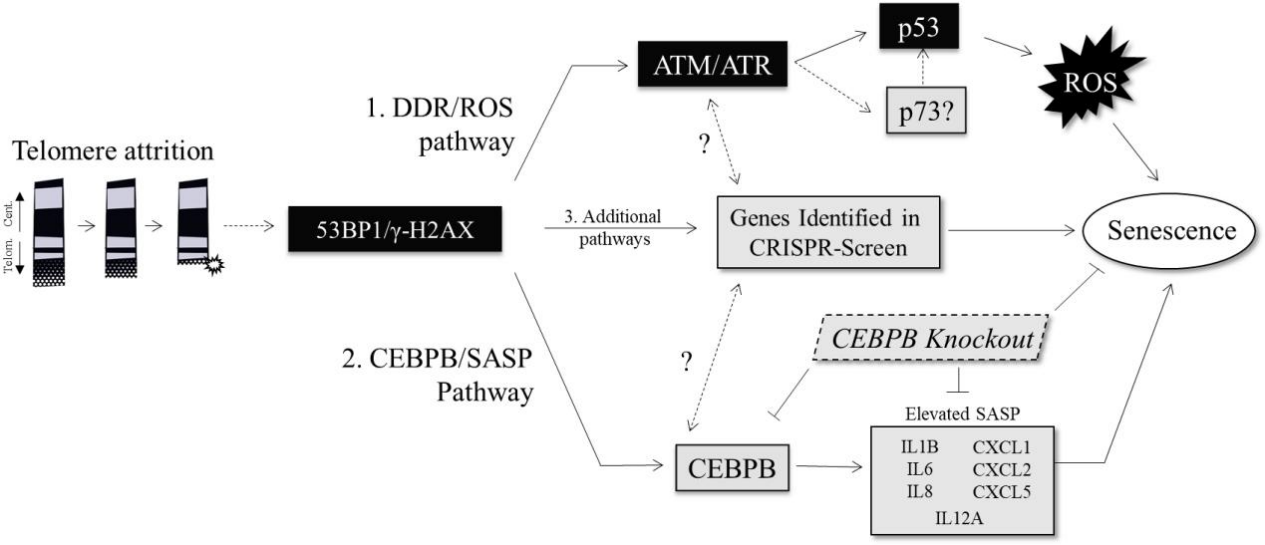
CEBPB increases inflammation/SASP independent of DDR pathway to promote senescence. Schematic of telomere-related senescence in DC cells. Deposition of 53BP1 and γ-H2AX is found on critically shortened telomeres leading to a DDR signaling pathway. This pathway activates ATM/ATR and p53 and ultimately leads to increased ROS and senescence. Alternatively, data supported by this research support the activation of a second pathway that includes mobilization of CEBPB and CEBPB-regulated genes related to SASP. When telomeres are elongated or CEBPB is knocked out, SASP gene expression is likely suppressed affording an extension of life span to these cells. Possible interplay between CEBPB, p73, and other genes found in the knockout screen are possible.

The recovery of CEBPB and other genes within our CRISPR screen indicates that additional mediators of senescence exist that may have not been previously appreciated. CEBPB and the downstream SASP response appear to be influenced by TERT activity and not DDR according to our p53 shRNA/ROS experiments. Although initiated by dysfunctional telomeres, the CEBPB and DDR/ROS pathways likely belong to independent but redundant pathways each contributing to senescence. A model for the relationship among shortened telomeres, DDR, and CEBPB/inflammation is presented in Fig. 5. Whether TP73, MED28, and WSB1 exist within this pathway or elsewhere is a question for future research. It is possible DC disease pathology could arise from an excessive or chronic inflammatory response. Limited therapeutic options exist in DC, and novel approaches are needed. Links have been found between inflammation and telomere dysfunction in mice (Jurk *et al*. 2014) and zebrafish (Lex *et al*. 2020), but future research will need to investigate inflammation in DC animal models and assess how pathogenic mutations impact various cell compartments and organ systems. If DC phenotypes do in fact arise as a result of CEBPB/SASP activation from short telomere signaling, manipulation of this pathway may be an interesting approach toward disease amelioration.

## Supplementary Material

jkad207_Supplementary_Data

## Data Availability

Cell lines are available upon request. RNA-seq has been archived online with GEO (GEO number: GSE224293). The authors affirm that all data necessary for confirming the conclusions of the article are present within the article, figures, and tables. The CEBPB gRNA deletion sequence is provided as Supplementary File 1. Differential expression sequencing results of DC, control, DC-shp53, and DC TERT are provided as Supplementary Files 2–4. Supplemental material available at G3 online.
