# Supplementary material for: CRISPR screen identifies CEBPB as contributor to dyskeratosis congenita fibroblast senescence via augmented inflammatory gene response
Source: G3 (Bethesda). 2023 Sep 17;13(11):jkad207. doi: 10.1093/g3journal/jkad207 (PMC10627266; doi:10.1093/g3journal/jkad207)
Supplement: jkad207_Supplementary_Data [file jkad207_supplementary_data.zip › Supplemental_Material_Legends_G3-2023-404396.docx]

**Supplementary File 1:**

CEBPB gRNA deletion sequence

**Supplemental File 2:**

Differential Expression Sequencing results of DC versus Control

**Supplemental File 3:**

Differential Expression Sequencing results of DC versus DC-shp53

**Supplemental File 4:**

Differential Expression Sequencing results of DC versus DC TERT
