# Supplementary material for: CRISPR screen identifies CEBPB as contributor to dyskeratosis congenita fibroblast senescence via augmented inflammatory gene response
Source: G3 (Bethesda). 2023 Sep 17;13(11):jkad207. doi: 10.1093/g3journal/jkad207 (PMC10627266; doi:10.1093/g3journal/jkad207)
Supplement: jkad207_Supplementary_Data [file jkad207_supplementary_data.zip › Supplementary_File_1_G3-2023-404396.docx]

**Supplemental File:**

1.CEBPB gRNA deletion sequence

CEBPB gRNA: TACGCGCTGCGCGCTTACCT

PCR sequence Verification: TTACTACGAGGCGGACTGCTTGGCTGCTGCGTACGGCGGCAAGGCGGDELETIONCCCCGACCGCCTGCTACGCGGGGGCCGCGCCGGCGCCCTCGCA

597bp deleted sequence: CCCCCGCGGCGCCCCCCGCGGCCAGACCCGGGCCGCGCCCCCCCGCCG

GCGAGCTGGGCAGCATCGGCGACCACGAGCGCGCCATCGACTTCAGCCCG

TACCTGGAGCCGCTGGGCGCGCCGCAGGCCCCGGCGCCCGCCACGGCCAC

GGACACCTTCGAGGCGGCTCCGCCCGCGCCCGCCCCCGCGCCCGCCTCCT

CCGGGCAGCACCACGACTTCCTCTCCGACCTCTTCTCCGACGACTACGGG

GGCAAGAACTGCAAGAAGCCGGCCGAGTACGGCTACGTGAGCCTGGGGCG

CCTGGGGGCCGCCAAGGGCGCGCTGCACCCCGGCTGCTTCGCGCCCCTGC

ACCCACCGCCCCCGCCGCCGCCGCCGCCCGCCGAGCTCAAGGCGGAGCCG

GGCTTCGAGCCCGCGGACTGCAAGCGGAAGGAGGAGGCCGGGGCGCCGGG

CGGCGGCGCAGGCATGGCGGCGGGCTTCCCGTACGCGCTGCGCGCTTACC

TCGGCTACCAGGCGGTGCCGAGCGGCAGCAGCGGGAGCCTCTCCACGTCC

TCCTCGTCCAGCCCGCCCGGCACGCCGAGCCCCGCTGACGCCAAGGCGC
